# Tumor‐suppressive roles of ΔNp63β‐miR‐205 axis in epithelial–mesenchymal transition of oral squamous cell carcinoma via targeting ZEB1 and ZEB2

**DOI:** 10.1002/jcp.26267

**Published:** 2018-05-10

**Authors:** Yuma Hashiguchi, Shintaro Kawano, Yuichi Goto, Kaori Yasuda, Naoki Kaneko, Taiki Sakamoto, Ryota Matsubara, Teppei Jinno, Yasuyuki Maruse, Hideaki Tanaka, Masahiko Morioka, Taichi Hattori, Shoichi Tanaka, Tamotsu Kiyoshima, Seiji Nakamura

**Affiliations:** ^1^ Section of Oral and Maxillofacial Oncology, Division of Maxillofacial Diagnostic and Surgical Sciences, Faculty of Dental Science Kyushu University Fukuoka Japan; ^2^ Maxillofacial Diagnostic and Surgical Science, Department of Oral and Maxillofacial Rehabilitation, Course for Developmental Therapeutics Kagoshima University Graduate School of Medical and Dental Sciences Kagoshima Japan; ^3^ Cell Innovator, Inc. Venture Business Laboratory of Kyushu University Fukuoka Japan; ^4^ Laboratory of Oral Pathology, Division of Maxillofacial Diagnostic and Surgical Sciences, Faculty of Dental Science Kyushu University Fukuoka Japan

**Keywords:** ΔNp63β, cancer, EMT, miRNA, OSCC

## Abstract

We previously revealed that epithelial‐to‐mesenchymal transition (EMT) was mediated by ΔNp63β, a splicing variant of ΔNp63, in oral squamous cell carcinoma (OSCC). Recent studies have highlighted the involvement of microRNA (miRNA) in EMT of cancer cells, though the mechanism remains unclear. To identify miRNAs responsible for ΔNp63β‐mediated EMT, miRNA microarray analyses were performed by ΔNp63β‐overexpression in OSCC cells; SQUU‐B, which lacks ΔNp63 expression and displays EMT phenotypes. miRNAs microarray analyses revealed miR‐205 was the most up‐regulated following ΔNp63β‐overexpression. In OSCC cells, miR‐205 expression was positively associated with ΔNp63 and negatively with zinc‐finger E‐box binding homeobox (ZEB) 1 and ZEB2, potential targets of miR‐205. miR‐205 overexpression by miR‐205 mimic transfection into SQUU‐B cells led to decreasing ZEB1, ZEB2, and mesenchymal markers, increasing epithelial markers, and reducing cell motilities, suggesting inhibition of EMT phenotype. Interestingly, the results opposite to this phenomenon were obtained by transfection of miR‐205 inhibitor into OSCC cells, which express ΔNp63 and miR‐205. Furthermore, target protector analyses revealed direct regulation by miR‐205 of ZEB1 and ZEB2 expression. These results showed tumor‐suppressive roles of ΔNp63β and miR‐205 by inhibiting EMT thorough modulating ZEB1 and ZEB2 expression in OSCC.

## 1 INTRODUCTION

Oral squamous cell carcinoma (OSCC) is one of the most common malignancies arising in oral cavity. Recent remarkable advancement of diagnostic modalities and reconstructive surgery led early detection of OSCC and radical operation for advanced cancer. However, the overall survival rate of OSCC patients has not risen satisfactory during last decade because of local aggressiveness and metastasis (Jerjes et al., 2010; Massano et al., 2006). Metastasis progresses through multi‐step process which includes local cancer invasion, entry into the vasculature followed by the exit of cancer cells from the circulation and colonization at the distal sites (Spano, Heck, De Antonellis, Christofori, & Zollo, 2012; Woodhouse et al., 1997). Therefore, to clarify the molecular mechanism of cancer invasion as a first step of metastasis leads to improve the survival rate of OSCC patients by inhibiting metastasis.

Epithelial‐to‐mesenchymal transition (EMT) is a biological process characterized by biochemical and morphological changes that enable epithelial cell to acquire a mesenchymal cell phenotype during embryonic development and wound healing (Hay, 1995). Following EMT, migratory capacity is enhanced and production of extracellular matrix is greatly increased (Thiery, 2002). Recent reports have revealed that EMT is involved in cancer invasion and progression (Potenta, Zeisberg, & Kalluri, 2008). It has also been demonstrated that Snail, a zinc finger transcription factor involved in cancer cell EMT, directly suppresses E‐cadherin expression (Batlle et al., 2000). Other zinc finger transcription factors, including zinc finger E‐box binding homeobox (ZEB) 1, Slug and Twist have also been reported to repress E‐cadherin expression and induce EMT (Adhikary et al., 2014; Sarrio et al., 2008; Yang et al., 2004). Previously, we have also studied on the association of p63 gene, a homolog of the p53 tumor suppressor gene, with EMT in OSCC cells (Goto et al., 2014; Matsubara et al., 2011). p63 has two different promoter domains that generate two protein isoforms, TAp63 and ΔNp63 (Levine, Tomasini, McKeon, Mak, & Melino, 2011). In addition, each isoform yields three isotypes (α, β, and γ) generated by alternative splicing of the p63 COOH terminus (Yang et al., 2004). TAp63 transactivates p53 target genes that induce apoptosis by inhibiting cell proliferation in response to exposure to DNA‐damaging agents (Crook, Nicholls, Brooks, O'Nions, & Allday, 2000). Conversely, ΔNp63 exerts dominant‐negative activities against TAp63 and p53, and is thus considered an oncoprotein (Higashikawa et al., 2009). Our previous data demonstrated that down‐regulation of ΔNp63 accompanied with EMT and that re‐expression of ΔNp63β by stable cDNA transfection in OSCC cell lines reverted the EMT phenotype (Goto et al., 2014). However, the detail mechanisms of ΔNp63β‐mediated EMT remain to be unclear.

microRNAs (miRNAs) are small non‐coding RNAs of 20–23 nucleotides in length that have a crucial role in post‐transcriptional regulation of gene expression by binding to a target site in the 3′‐UTR of target mRNAs (Ambros, 2004). miRNAs are reported to regulate the expression of genes that mediate critical processes in tumorigenesis, such as differentiation, cell cycle regulation, apoptosis, and invasion (Esquela‐Kerscher & Slack, 2006; Farazi, Hoell, Morozov, & Tuschl, 2013). Recent studies have shown that several miRNAs are also involved in EMT process of human cancer by targeting EMT‐related transcription factors (Gregory, Bracken, Bert, & Goodall, 2008; Zaravinos, 2015). We thus sought to identify responsible miRNA associated with ΔNp63β‐mediated EMT using miRNA microarray analyses in this study. As samples for miRNA microarray, we used two clones established from an OSCC cell line SQUU‐B, which lacks ΔNp63 expression and displays EMT phenotypes. One is SQUU‐BO cell overexpressing ΔNp63β, and another is SQUU‐BC cell transfected with empty vector (Goto et al., 2014). By comparing expression profiles of the two OSCC clones, we focused on miR‐205, which is involved in epithelial differentiation (Ryan, Oliveira‐Fernandes, & Lavker, 2006), because miR‐205 expression was remarkably increased in SQUU‐BO cells. Herein, we present some evidences that miR‐205 is possibly involved in ΔNp63β‐mediated EMT.

## 2 MATERIALS AND METHODS

### 2.1 Cell cultures experiments

This study was approved by the Ethics Committee of Kyushu University hospital (IRB serial number: 27–362). Seven OSCC cell lines (HSC‐2, HSC‐3, SQUU‐A, SQUU‐B, SQUU‐BO, SQUU‐BC, and SAS) and a keratinocyte cell (HaCaT) were used in this study. SQUU‐A and SQUU‐B cells were previously established from the same patient with recurrent tongue carcinoma by orthotopic implantation. SQUU‐B cells show histologically invasive growth and have high metastatic potential (86.7% incidence of cervical lymph node metastasis), whereas SQUU‐A cells demonstrate expansive growth and have low metastatic ability (Morifuji, Taniguchi, Sakai, Nakabeppu, & Ohishi, 2000). SQUU‐BO cells were generated by transfection with ΔNp63β expression vector, as previously described (Goto et al., 2014). All cell lines were maintained in a humidified atmosphere of 5% CO_2_ at 37 °C, and cultured in Dulbecco's modified Eagle's medium (DMEM)/F‐12 (Life Technologies, Carlsbad, CA) supplemented with 10% fetal bovine serum (FBS) and 100 units/ml penicillin/streptomycin.

### 2.2 miRNA microarray analyses

To find miRNA associated with ΔNp63β‐mediated EMT, total RNA including miRNA was extracted from SQUU‐BO and SQUU‐BC cells. The 100 ng of total RNA from each sample was labeled using FlashTag™ Biotin HSR RNA Labeling Kit (Affymetrix, Santa Clara, CA), and hybridized to a Affymetrix GeneChip^®^ miRNA 4.0 Array according to the manufacturer's instructions. All hybridized microarray slides were scanned using an Affymetrix scanner. Relative hybridization intensities and background hybridization values were calculated using Affymetrix Expression Console™.

### 2.3 Data analyses and filter criteria

We processed the raw CEL files for gene‐level analysis with median polish summarization and quantile normalization by Affymetrix^®^ Transcriptome Analysis Console Software, and obtained normalized intensity values. To identify up or down‐regulated genes, we calculated ratios (non‐log scaled fold‐change) from the normalized intensities of each gene for comparisons between SQUU‐BC and SQUU‐BO cells. Then we established criteria for regulated genes with average signal levels of either more than 100: (up‐regulated genes) ratio ≥2.0‐fold, (down‐regulated genes) ratio ≤0.5. In addition, prediction of target genes of miRNAs was performed using miRTarBase (http://mirtarbase.mbc.nctu.edu.tw; September 2015 released) (Chou et al., 2016) and MicroCosm (http://www.ebi.ac.uk/microcosm) (Griffiths‐Jones, Saini, van Dongen, & Enright, 2008) software which is a comprehensive resource of miRNA targets and expression profiles.

### 2.4 RNA extraction and complementary DNA (cDNA) synthesis

For mRNA analysis, total RNA was extracted from cultured cells using TRIzol reagent (Invitrogen, Carlsbad, CA). The amount of RNA extracted from each sample was measured spectrophotometrically (NANO DROP 1000, Thermo Fisher Scientific, Waltham, MA). Two micrograms of the total RNA preparation was then used to synthesize cDNA. Briefly, RNA was incubated for 15 min at 42 °C with 35 units/µl of recombinant RNase inhibitor (Nacalai Tesque, Kyoto, Japan), 1.0 µl of 50 µM random hexamers (Invitrogen), 2.0 µl of each 2.0 mM dNTP (Toyobo, Osaka, Japan), and 50 units/µl of Murine leukemia virus (MuLV) reverse transcriptase (Applied Biosystems, Foster City, CA). For miRNA analysis, total RNA was extracted using a miRNeasy Mini Kit (QIAGEN, Hilden, Germany), and reverse transcription was performed using miScript II RT Kit (QIAGEN) according to the manufacture's instructions.

### 2.5 Quantitative real‐time PCR

Real‐time PCR was performed for quantification of gene expression levels, using Brilliant II SYBR Green QPCR Master Mix (Agilent Technologies, Santa Clara, CA). Gene expression levels were quantified using the Real‐time PCR System (Mx3000P, Stratagene, San Diego, CA), according to the manufacturer's instructions. For amplification of specific regions of target genes, the primers used were listed in Table 1. For miRNA detection, real‐time PCR was performed with a miScript SYBR Green PCR Kit (QIAGEN). The primers for miRNAs were as follows: miR‐205 (hsa‐miR‐205‐5p, MS00003780, QIAGEN), and RNU6B (U6 small nuclear RNA 2, MS00033740, QIAGEN). For relative quantification, the 2^−ΔΔCt^ method, representing fold changes in the target genes normalized to a reference gene, was used. Glyceraldehyde‐3‐phosphate dehydrogenase (GAPDH) and RNU6B were used as the internal control.

**Table 1:** Primers used in this study

mRNA	Size (bp)		Sequence
ΔNp63	117	Forward	TGCCCAGACTCAATTTAGTGAG
		Reverse	TGCGCGTGGTCTGTGTTATA
ZEB1	118	Forward	AAGACATGTGACGCAGTCTGG
		Reverse	TGGCTTCTCTCCACTGTGAATTC
ZEB2	211	Forward	AGCGGAAACAAGGATTTC
		Reverse	GGTCTTTTTCCTGTGTGTTCG
E‐cadherin	196	Forward	TGCTCTTCCAGGAACCTCTG
		Reverse	AGGGAAACTCTCTCGGTCCA
CK19	174	Forward	TCCGAACCAAGTTTGAGACG
		Reverse	TGATTTCCTCCTCATGGTTGTT
Vimentin	196	Forward	TGCCCTTAAAGGAACCAATG
		Reverse	CTCAATG TCAAGGGCCATCT
N‐cadherin	163	Forward	TGAAGGAGTCAGCAGAAGTTGA
		Reverse	TCAGACCTGATCCTGACAAGC
Fibronectin	170	Forward	GCAAGCCCATAGCTGAGAAG
		Reverse	GTCCTGATCGTTGCATCTATTT C
GAPDH	104	Forward	ATCAGCAATGCCTCCTGCAC
		Reverse	ATGGCATGGACTGTGGTCAT

### 2.6 Immunocytochemistry

Cultured cells were fixed in 75% methanol and then incubated with each primary antibody. The primary antibodies used were shown in Table 2. Subsequently, the cells were incubated with secondary antibodies conjugated with anti‐mouse or rabbit fluorescence‐labeled antibody (Alexa Fluor® 488 and 546, Molecular Probes, Eugene, OR; diluted 1:400). The cells were counterstained with DAPI (Vector Laboratories, Burlingame, CA). Fluorescently‐labeled cells were observed under a fluorescence microscope (BZ‐9000, KEYENCE, Tokyo, Japan).

**Table 2:** Primary antibodies used in this study

Antibodies	Application	Dilution
Polyclonal rabbit anti‐human ZEB1 antibodies (Atlas Antibodies AB, Stockholm, Sweden)	ICC	1/200
Polyclonal rabbit anti‐human ZEB2 antibodies (Abcam, Cambridge, UK)	ICC	1/200
Monoclonal mouse anti‐human E‐cadherin antibody (BD biosciences, Franklin Lakes, NJ)	ICC	1/200
	WB	1/1000
Monoclonal rabbit anti‐human ZEB1 antibody (Cell Signaling Technology, Danvers, MA)	WB	1/500
Polyclonal rabbit anti‐human ZEB2 antibodies (Merck Millipore, Darmstadt, Germany)	WB	1/2000
Monoclonal mouse anti‐human β‐actin antibody (C4, Santa Cruz Biotechnology, Dallas, TX)	WB	1/1000

ICC, immunocytochemical staining; WB, Western blotting.

### 2.7 Western blotting

Cell lysate was denatured, separated by sodium dodecyl sulfate–polyacrylamide gel electrophoresis (SDS‐PAGE), and transferred onto a polyvinylidene difluoride (PVDF) membrane (Millipore, Billerica, MA). The PVDF membrane was incubated with blocking buffer and then incubated separately with primary antibodies at 4 °C overnight, followed by horseradish peroxidase (HRP)‐conjugated secondary antibodies (Jackson ImmunoResearch, West Grove, PA) at room temperature for 1 hr. The primary antibodies used were shown in Table 2. The detection of specific proteins was carried out with enhanced chemiluminescence reagents (Chemi‐Lumi One Super, Nacalai Tesque) and visualized using ImageQuant LAS 4000 (Fuji Film, Tokyo, Japan). β‐actin was used as a positive control.

### 2.8 Small interfering RNA (siRNA) transfection

The sequences of the siRNAs used in this study were as follows: ΔNp63 siRNA, 5′‐GGACAGCAGCATTGATCAATT‐3′, scrambled siRNA, 5′‐CAGTCGCGTTTGCGACTGG‐3′ (Sigma–Aldrich, St. Louis, MO). Cells were seeded at 2 × 10^5^ cells per well in six‐well plates and transfected with siRNAs using Lipofectamin RNAi MAX reagent (Invitrogen). Extraction of RNA was performed 48 hr after transfection.

### 2.9 miRNA mimic and inhibitor transfection

Cells were seeded at 2 × 10^5^ cells per well in six‐well plates and transfected with miScript miR‐205 mimic (Syn‐hsa‐miR‐205‐5p: MSY0000266, QIAGEN), miScript miR‐205 inhibitor (Anti‐hsa‐miR‐205‐5p: MIN0000266, QIAGEN), or each control miRNA (QIAGEN). All miRNA mimic, miRNA inhibitor, and control miRNA were transiently transfected into cells using Lipofectamine RNAiMAX (Invitrogen) according to the manufacturer's instructions. Extraction of RNA and proteins were performed 48 hr and 72 hr after transfection, respectively.

### 2.10 Target inhibition analyses of miRNA

We predicted the miR‐205‐binding sites in the 3′‐UTR of target gene by TargetScan (http://www.targetscan.org; August 2010 released) software (Agarwal, Bell, Nam, & Bartel, 2015; Lewis et al., 2005), and the specific complimentary sequence for the target site was synthesized using miScript Target Protector (QIAGEN). miR‐205 mimic was co‐transfected with the target or control protector in the SQUU‐B cells according to the manufacturer's instructions. At 72 hr after transfection, cells were harvested for protein extraction.

### 2.11 Wound healing assay

Cells were seeded in 24‐well culture dishes and transfected. After 48 hr of the transfection, a wound was incised with a pipette tip in the central area of the confluent culture on the dishes. In order to inhibit cell proliferation, mitomycin C was added to cell cultures at 10 µg/ml for 2 hr after scratching. Detached cells were removed carefully with PBS and migration of cells into the wound areas was observed using a phase‐contrast microscope (CKX41 NB‐31PHP, Olympus, Tokyo, Japan). The area reduction rates were then calculated.

### 2.12 Matrigel™ invasion assay

Cells were seeded at a density of 2 × 10^5^/well on 60‐mm dishes, and then the cells were transfected with miR‐205 mimic, miR‐205 inhibitor, or control miRNA. After 48 hr, cells were loaded in the upper well of the BioCoat™ Matrigel™ invasion chambers (BD Biosciences, Franklin Lakes, NJ) and equal number of the control inserts (BD Biosciences). The cells were incubated for 22 hr and then the invaded cells on the outside of the inserts were visualized using hematoxylin and eosin (HE). The number of invaded cells was counted under a light fluorescence (BZ‐9000, KEYENCE). The evaluation of invasion was carried out by percent invasion. The percent invasion was calculated as follows: (mean of number of cells invading through Matrigel™ insert membrane per mean of number of cells migrating through control insert membrane) × 100 (%).

### 2.13 Water‐soluble tetrazolium (WST)‐8 cell proliferation assay

Cell proliferation assays were performed using the Cell Count Reagent SF (Nacalai Tesque), according to the manufacturer's instructions. Cells were seeded at a density of 2 × 10^5^/well on 60‐mm dishes, and then the cells were transfected with miR‐205 mimic, miR‐205 inhibitor, or control miRNA. After 48 hr, the transfected cells were replaced 2.0 × 10^3^ cells/well into 96‐well plates. After 12 hr of incubation, 10 µl of premixed reagent was added to each well. The plates were further incubated for 2 hr, and absorbance at 450 nm was then measured using a microplate reader (MULTISKAN FC, Thermo Fisher Scientific).

### 2.14 Statistical analyses

All statistical analyses were performed with JMP software version 11 (SAS Institute, Tokyo, Japan). The Mann–Whitney U‐test was also used to compare relative mRNA and miRNA expression levels by real‐time PCR methods. It was also used to assess the significance of the differences between each group in the wound healing assay, cell proliferation assay, and invasion assay. A *p*‐value of less than 0.05 was considered statistically significant.

## 3 RESULTS

### 3.1 Identification of the miRNAs involved in ΔNp63β‐mediated EMT in OSCC cells

**Figure 1:**
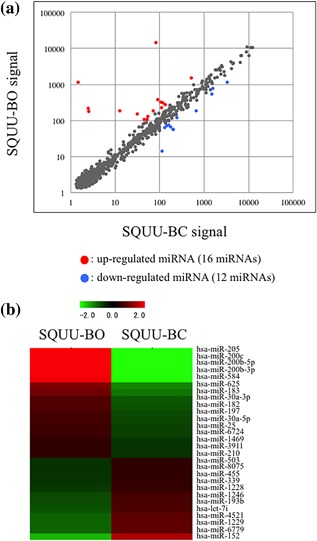
Differential expression profiling by ΔNp63β‐overexpression in miRNA microarray analyses. (a and b) Scatterplot analysis and heat map visualize the differences of miRNAs expression profiles between SQUU‐BO cells, which overexpressed ΔNp63ß, and SQUU‐BC cells transfected empty vector. (a) Scatterplot analysis. Red dots show up‐regulated miRNAs and blue dots show down‐regulated. (b) Heat map analysis. Red color shows higher expression levels and green shows lower

To identify the responsible miRNAs involved in ΔNp63β‐mediated EMT, miRNA microarray analyses were performed by using SQUU‐BC and SQUU‐BO cells. miRNA microarray analyses revealed that 16 miRNAs were significantly up‐regulated and 12 miRNAs were down‐regulated in SQUU‐BO cells compared with SQUU‐BC cells among 6599 miRNAs (see Table 3). The heat map and scatterplot showed differentially expressed miRNAs between two clones (Figure 1). In the results of miRNA microarray analyses, miR‐205 was remarkably overexpressed in SQUU‐BO cells compared with SQUU‐BC cells. We thus focused on miR‐205 for the potential target involved in ΔNp63β‐mediated EMT.

**Table 3:** Upregulated or down‐regulated miRNA from the total 6599 probes

	miRNA	miRBase acc no.	Fold change
Up‐regulated			
1	hsa‐miR‐205	MIMAT0000266	786.88
2	hsa‐miR‐200c	MIMAT0000617	176.07
3	hsa‐miR‐200b‐5p	MIMAT0004571	86.82
4	hsa‐miR‐200b‐3p	MIMAT0000318	71.51
5	hsa‐miR‐584	MIMAT0003249	14.83
6	hsa‐miR‐625	MIMAT0003294	4.96
7	hsa‐miR‐183	MIMAT0000261	4.23
8	hsa‐miR‐30a‐3p	MIMAT0000088	3.05
9	hsa‐miR‐182	MIMAT0000259	2.95
10	hsa‐miR‐197	MIMAT0000227	2.64
11	hsa‐miR‐30a‐5p	MIMAT0000087	2.58
12	hsa‐miR‐25	MIMAT0004498	2.41
13	hsa‐miR‐6724	MIMAT0025856	2.36
14	hsa‐miR‐1469	MIMAT0007347	2.06
15	hsa‐miR‐3911	MIMAT0018185	2.06
16	hsa‐miR‐210	MIMAT0000267	2.04
Down‐regulated			
1	hsa‐miR‐152	MIMAT0000438	0.12
2	hsa‐miR‐6779	MIMAT0027458	0.28
3	hsa‐miR‐1229	MIMAT0022942	0.28
4	hsa‐miR‐4521	MIMAT0019058	0.29
5	hsa‐let‐7i	MIMAT0000415	0.34
6	hsa‐miR‐193b	MIMAT0002819	0.36
7	hsa‐miR‐1246	MIMAT0005898	0.37
8	hsa‐miR‐1228	MIMAT0005582	0.46
9	hsa‐miR‐339	MIMAT0000764	0.49
10	hsa‐miR‐455	MIMAT0004784	0.49
11	hsa‐miR‐8075	MIMAT0031002	0.5
12	hsa‐miR‐503	MIMAT0002874	0.5

### 3.2 Expression of miR‐205 in OSCC cells

**Figure 2:**
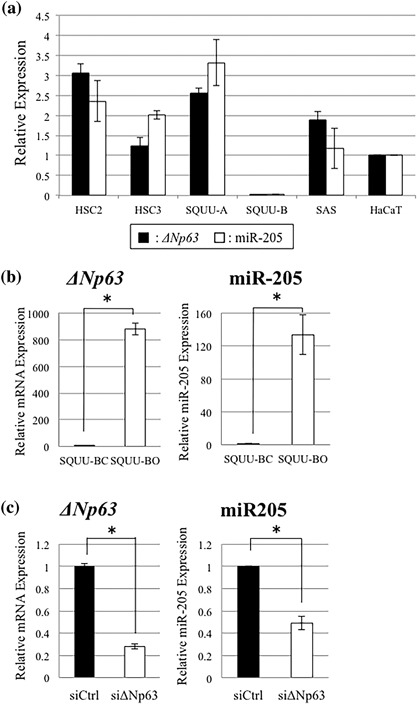
Expression levels of ΔNp63 and miR‐205 in the OSCC cell lines. (a) Real‐time PCR analysis demonstrates that expression levels of *ΔNp63* are positively associated with miR‐205, and most weakly in the SQUU‐B cells, which display EMT phenotype. (b) Real‐time PCR analyses demonstrate that miR‐205 expression level in SQUU‐BO cells is higher than SQUU‐BC cells (Mann–Whitney U‐test, *p < 0.05). (c) miR‐205 expression level is significantly down‐regulated in the ΔNp63‐knockdown cells compared with control cells (Mann–Whitney U‐test, *p < 0.05)

Real‐time PCR analyses were performed to examine the expression levels of ΔNp63 and miR‐205 in OSCC cell lines. miR‐205 expression level was most strongly in the SQUU‐A and weakly in the SQUU‐B, which lacks ΔNp63 expression (Figure 2a). Furthermore, the expression level of miR‐205 was positively correlated with ΔNp63 expression. In the SQUU‐BO overexpressing ΔNp63β, miR‐205 expression was also higher than that in control cells (Figure 2b). To further investigate the association between miR‐205 and ΔNp63 expression, we transfected ΔNp63 siRNA into SQUU‐A cells. Following ΔNp63 knockdown, the expression of miR‐205 was significantly decreased than that in control cells (Figure 2c).

### 3.3 Expression of ZEB1 and ZEB2, miR‐205 targeting genes, in the OSCC cells

**Figure 3:**
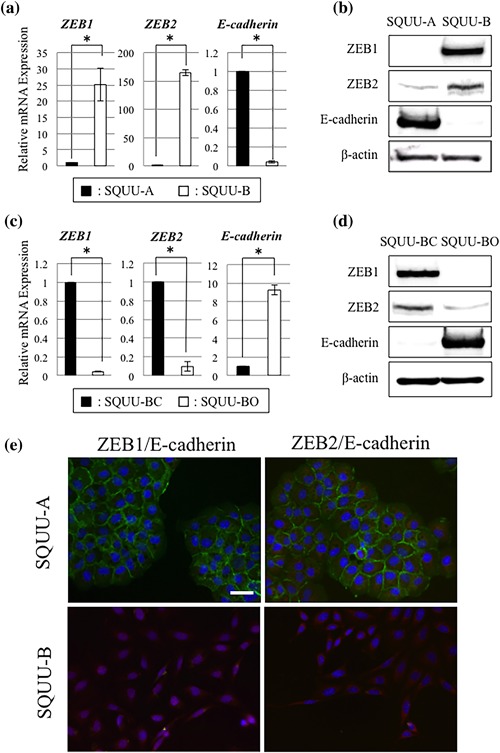
Expression of *ZEB1*, *ZEB2*, and *E‐cadherin* in the OSCC cells. (a and b) Real‐time PCR and Western blot analyses demonstrate that SQUU‐B cells with low expression levels of ΔNp63 and miR‐205 show higher expression of ZEB1 and ZEB2 and lower expression of E‐cadherin compared with SQUU‐A cells, which expresses ΔNp63 and miR‐205 (Mann–Whitney U‐test, * *p *< 0.05). (c and d) Following ΔNp63 overexpression, expression of ZEB1 and ZEB2 are down‐regulated, whereas expression level of E‐cadherin is elevated (Mann–Whitney U‐test, * *p* < 0.05). (e) Double staining of ZEB1 or ZEB2 with E‐cadherin in OSCC cells by immunocytochemistry. The strong expressions of ZEB1 and ZEB2 are observed mainly in the nuclei of SQUU‐B cells, but E‐cadherin not found. On the other hand, the expression of E‐cadherin is detected in the cellular membrane of SQUU‐A cells, but expression of ZEB1 and ZEB2 is undetectable. Green, E‐cadherin; Red, ZEB1 or ZEB2. Scale bar; 20 µm

To identify the target genes of miR‐205, we used miRTarBase and MicroCosm software and further analyzed. Among the candidate genes targeted by miR‐205, we focused on ZEB1 and ZEB2 (SIP1 and ZFHX1B) that have been reported as EMT‐related transcription factors (Wong, Gao, & Chan, 2014). ZEB1 and ZEB2 have been shown to directly bind to the E‐cadherin promoter and repress its transcription. We thus examined the expression of ZEB1, ZEB2, and E‐cadherin in OSCC cells by real‐time PCR and Western blot analyses. Compared with SQUU‐A cells with high expression levels of miR‐205, ZEB1 and ZEB2 expression was stronger in SQUU‐B cells, while the expression of E‐cadherin was weaker (Figures 3a and 3b). Conversely, SQUU‐BO cells showed the weaker expression of ZEB1 and ZEB2 and stronger expression E‐cadherin compared with control cells. (Figures 3c and 3d).

Furthermore, we elucidated the localization of ZEB1, ZEB2, and E‐cadherin in OSCC cells by immunocytochemical double staining. The expression of ZEB1 and ZEB2 was observed strongly in the nucleus and slightly in the cytoplasm of SQUU‐B cells, but E‐cadherin was undetectable. On the other hand, in SQUU‐A cells, though strong immunoreactivities for E‐cadherin was found, ZEB1 and ZEB2 expression was undetectable (Figure 2e).

### 3.4 Effects of miR‐205 overexpression on EMT phenotype of OSCC cells

**Figure 4:**
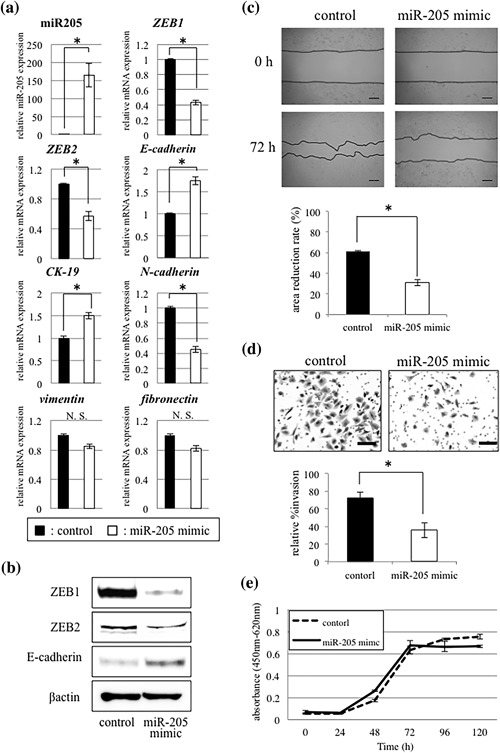
Effects of miR‐205 mimic transfection in SQUU‐B cells. (a and b) Expression of ZEB1 and ZEB2 is decreased and E‐cadherin increased in the cells transfected with miR‐205 mimic. Up‐regulation of *CK19* and down‐regulation of *N‐cadherin* are also seen (Mann–Whitney U‐test, *p < 0.05). However, there are no significant differences in the expression levels of *vimentin* and *fibronectin*. (c) Wound healing assay demonstrates that cell migration capacity is significantly inhibited in miR‐205‐overexpressing OSCC cells, compared with control cells at 72 hr. (Mann–Whitney U‐test, * *p *< 0.05). Scale bars; 200 µm. (d) miR‐205‐overexpressing cells show low invasion ability compared with control cells in Matrigel™ invasion assay (Mann–Whitney U‐test, *p < 0.05). Scale bars; 50 µm. (e) WST‐8 assay shows that there is no significant difference in the cell proliferation between miR‐205‐overexpressing cells and control cells (Mann–Whitney U‐test)

To determine the functional roles of miR‐205 in ΔNp63β‐mediated EMT, miR‐205 mimic was transfected into SQUU‐B cells for overexpressing miR‐205. As a result of miR‐205 overexpression, ZEB1 and ZEB2 were significantly decreased at both the gene and protein levels, while those of E‐cadherin were increased (Figures 4a and 4b). Although up‐regulation of cytokeratin (CK) 19 and down‐regulation of N‐cadherin (Figure 4a) were also found, expression of vimentin and fibronectin remained unaffected.

In the wound healing assay and Matrigel™ invasion assay, SQUU‐B cells transfected with miR‐205 mimic showed significantly lower migration and invasion abilities compared with the control cells (Mann‐Whiteney U‐test, *p* < 0.05) (Figures 4c and 4d). However, in the WST‐8 assay, no significant difference in the cell proliferation was observed between the cells with overexpression of miR‐205 and without (Figure 4e).

### 3.5 Effects of miR‐205 knockdown on EMT phenotype of OSCC cells

**Figure 5:**
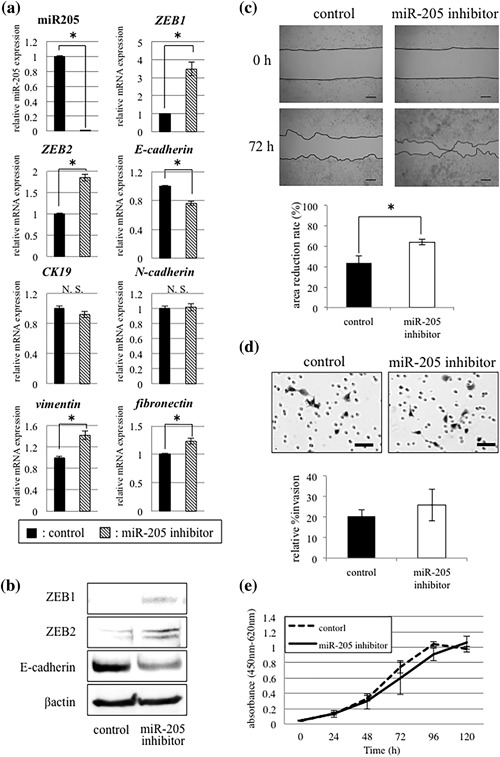
Effects of miR‐205 inhibitor transfection in SQUU‐A cells. (a and b) Real‐time PCR and Western blot analyses demonstrate that knockdown of miR‐205 leads to up‐regulation of ZEB1 and ZEB2 and down‐regulation of E‐cadherin. Up‐regulation of *vimentin* and *ficronectin* are also observed (Mann–Whitney U‐test, * *p *< 0.05). On the other hand, there are no significant differences in *CK19* and *N‐cadherin*. (c) Wound healing assay demonstrates that knockdown of miR‐205 enhances migration capacity compared with control cells at 72 h (Mann–Whitney U‐test, * *p *< 0.05). Scale bars; 200 µm. (d) Matrigel™ invasion assay shows high invasion ability in the cells with transfection of miR‐205 inhibitor compared with control cells, though no significant difference is found (Mann–Whitney U‐test). Scale bars; 50 µm. (e) WST‐8 assay shows no significant difference in cell proliferation between cells with silencing of miR‐205 and without (Mann–Whitney U‐test)

We further investigated influences of miR‐205 knockdown in OSCC cells by miR‐205 inhibitor transfection into SQUU‐A cells. Real‐time PCR and western blot analyses showed that ZEB1 and ZEB2 expression were elevated, while expression of E‐cadherin was decreased following transfection of miR‐205 inhibitor (Figure 5a and 5b). Moreover, up‐regulation of mesenchymal markers including vimentin and fibronectin were also found (Figure 5a).

In the wound healing assay, migration ability of the SQUU‐A cells transfected with miR‐205 inhibitor was promoted, compared with the control cells (Figure 5c). In the Matrigel™ invasion assay, the number of invaded cells was increased in the SQUU‐A cells with knockdown of miR‐205, compared with control cells, though no significant difference was shown (Figure 5d). In the WST‐8 assay, knockdown of miR‐205 did not affect on the proliferation of SQUU‐A cells (Figure 5e).

### 3.6 Interfering of miR‐205‐binding sites in ZEB1 or ZEB2 mRNA

**Figure 6:**
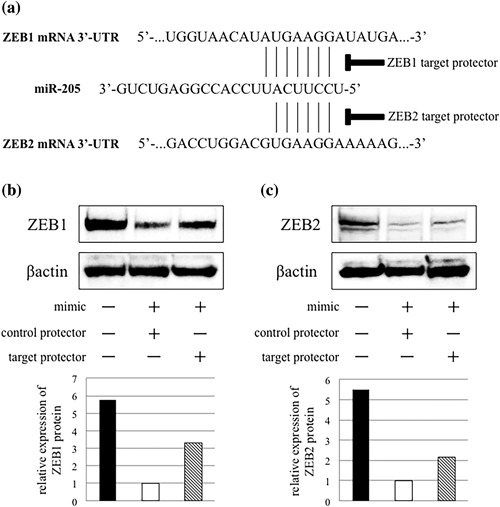
Interfering of miR‐205‐binding sites in ZEB1 or ZEB2 mRNA. (a) The figure shows miR‐205‐binding sites in ZEB1 and ZEB2 mRNA 3′‐UTR. The specific complimentary sequence for the target site is synthesized for this analysis. (b and c) Western blot analyses shows that co‐transfection of miR‐205 mimic with each target protector inhibits down‐regulation of ZEB1 and ZEB2 protein expression

The results in this study suggested that miR‐205 regulates ZEB1 and ZEB2 expression in OSCC cells. However, it remains unclear whether miR‐205 directly regulates these expressions. Therefore, we predicted the binding sites of miR‐205 in the 3′UTR of ZEB1 and ZEB2 mRNA by TargetScan software, generated the single strand RNAs as protectors, and performed target inhibition analyses (Figure 6a). By the co‐transfection of miR‐205 mimic and ZEB1 or ZEB2 target protector into SQUU‐B cells, the expression level of ZEB1 or ZEB2 proteins was recovered, though ZEB1 and ZEB2 expression was decreased when miR‐205 mimic and control protector were co‐transfected. (Figures 6b and 6c).

## 4 DISCUSSION

In our previous study, we showed that down‐regulated vimentin and down‐regulated E‐cadherin expression was found in the oral cancer cells at the invasive front. Interestingly, the vimentin positive rate or the presence of decreased intensity of ΔNp63 was positively associated with the frequencies of metastases and poor prognosis in the OSCC patients (Goto et al., 2014). These results indicated that ΔNp63 down‐regulation in cancer cells is associated with mesenchymal phenotype in tumor progression of OSCC. A few studies have demonstrated association between the ΔNp63 expression and EMT in squamous cell carcinoma (SCC). Higashikawa et al. (2007) demonstrated that Snail down‐regulated ΔNp63α, the predominant ΔNp63 isoform in SCC, thereby leading to induction of EMT. They also revealed that ΔNp63α regulated the inhibitor of differentiation‐3 (Id‐3), a dominant negative regulator of E2A that functions as a transcriptional repressor of E‐cadherin (Higashikawa et al., 2009). These results suggest that ΔNp63α expression is strongly associated with the acquisition of a mesenchymal phenotype. Similar to these findings, we previously demonstrated that ΔNp63β, a splicing variant of ΔNp63, is associated with the induction of EMT in OSCC (Goto et al., 2014). However, the precise molecular mechanism underlying the ΔNp63β‐mediated EMT in OSCC cells remains unclear. Recent studies have highlighted the involvement of miRNA in EMT of cancer cells (Zaravinos, 2015). In this study, we thus focused on miR‐205, remarkably increased in OSCC cells overexpressed ΔNp63β by miRNA microarray analyses.

miR‐205 is transcribed from the MIR205HG gene that is located in the second intron of LOC642587 locus in chromosome 1 (Lim, Glasner, Yekta, Burge, & Bartel, 2003). It has already reported that miR‐205 promotes human epidermal keratinocytes and corneal epithelial keratinocytes via the lipid phosphatase SHIP2 (Yu et al., 2010). On the other hands, growing evidences show that miR‐205 plays a key role in a variety of tumors and functions as an oncogene or a tumor suppressor gene determined by the cancer context or its target genes (Qin et al., 2013). In gynecological malignancies, miR‐205 was remarkably up‐regulated in endometrial carcinoma tissues, and enhance tumor proliferation and invasion (Jin & Liang, 2015). Li et al. (2015) showed miR‐205 up‐regulation induced cell proliferation and invasion in ovarian cancer. On the other hand, miR‐205 exerted tumor‐suppressive roles in some kinds of cancers including breast cancer, gastric cancer, and prostate cancer (Majid et al., 2010; Wu, Zhu, & Mo, 2009; Yin et al., 2014). As just described above, expression and functions of miR‐205 in cancer is controversial, because no distinct evidence for a role of miR‐205 as an oncogenic or tumor‐suppressive miRNA has been shown. The present study revealed that miR‐205 played a tumor‐suppressive role by inhibiting migration and invasion in OSCC.

Previously, several studies demonstrated miR‐205 expression and the significance as a biomarker in the head and neck squamous cell carcinoma (HNSCC) (Tran et al., 2007; Kimura et al., 2010). miR‐205 was firstly identified as an up‐regulated miRNA in HNSCC by microarray analysis (Tran et al., 2007). After that, Kimura et al. (2010) also demonstrated that miR‐205 could be a specific biomarker for HNSCC. Although there were a few studies on function of miR‐205 in HNSCC, most of these studies reported that miR‐205 suppresses oncogenic activities such as anti‐apoptosis and proliferation (Kim et al., 2013, 2014). Interestingly, Childs et al. (2009) showed that low‐level expression of miR‐205 significantly associated with loco‐regional recurrence independent of disease severity. They also described that combined low expression levels of miR‐205 and let‐7d, which target the Ras oncogene, are significantly correlated with poor prognosis in HNSCC. These data indicate close association of low‐level miR‐205 expression with cancer progression. In this study, miR‐205 knockdown led to elevated cell motility in OSCC cells, suggesting that our result may be supportive for Childs's data.

We further showed that enhancing cell motility by miR‐205 knockdown was associated with ΔNp63β‐mediated EMT. Recent study demonstrated that loss of ΔNp63 and miR‐205 enhanced cell migration and metastasis via targeting expression of ZEB1 in prostate cancer (Tucci et al., 2012). Furthermore, ΔNp63α‐mediated expression of miR‐205 contributed to the regulation EMT in bladder cancer cells, and that miR‐205 prevented EMT through suppression of ZEB1 and ZEB2 (Tran et al., 2013). Matsushima et al. (2011) also reported that miR‐205 modulates migration and invasion by regulating ZEB2 expression in esophageal SCC. Using miRNA target prediction algorithms, ErB3, E2F5, ZEB1, ZEB2, and protein kinase Cϵ have been identified as putative miR‐205 targets (Gandellini et al., 2009). In fact, knockdown of miR‐205 or ΔNp63 led to up‐regulation of ZEB1 and ZEB2 in OSCC cells, and the expression level of ZEB1 or ZEB2 was recovered by the co‐transfection of miR‐205 mimic and ZEB1 or ZEB2 target protector into SQUU‐B cells in this study. Previous studies employing a reporter assay confirmed miR‐205 binding to the each 3′‐UTR of ZEB1 or ZEB2 (Matsushima et al., 2011; Niu, Shen, Zhang, Zhao, & Lu, 2015). Together, these data demonstrated that miR‐205 directly inhibits ZEB1 or ZEB2 in OSCC cells.

As described above, it was revealed that ΔNp63α mediated miR‐205 expression in bladder cancer (Tran et al., 2013). In this study, however, the miR‐205 expression is regulated by not ΔNp63α but ΔNp63β. Previously, different effects of the three isoforms of ΔNp63 were showed by gene expression profiling of HNSCC cells overexpressing each ΔNp63 isoform (Boldrup, Coates, Gu, & Nylander, 2009). Thereby, ΔNp63β was most efficient activator of gene expression than ΔNp63α and ΔNp63γ despite low expression levels in HNSCC, whereas ΔNp63γ was most effective at repressing gene expression. These results thus indicated that miR‐205 expression was induced by both ΔNp63α and ΔNp63β.

In conclusion, we clarify that ΔNp63β regulates miR‐205 and that these effects contribute to EMT suppression through inhibiting ZEB1 and ZEB2 expression. To the best of our knowledge, this is the first report that elucidated the association of miR‐205 with ΔNp63β‐mediated EMT. Elucidating the functions of miR‐205 in ΔNp63β‐mediated EMT will facilitate the identification of valuable biomarkers of tumor recurrence and metastasis and the development of novel OSCC treatment.

## CONFLICTS OF INTEREST

All authors declare no conflicts of interest.
